# *Moraxella osloensis *Gene Expression in the Slug Host *Deroceras reticulatum*

**DOI:** 10.1186/1471-2180-8-19

**Published:** 2008-01-28

**Authors:** Ruisheng An, Srinand Sreevatsan, Parwinder S Grewal

**Affiliations:** 1 Entomology Department, The Ohio State University, Wooster, OH 44691, USA; 2 Veterinary Population Medicine Department, University of Minnesota, St. Paul, MN 55108, USA

## Abstract

**Background:**

The bacterium *Moraxella osloensis *is a mutualistic symbiont of the slug-parasitic nematode *Phasmarhabditis hermaphrodita*. In nature, *P. hermaphrodita *vectors *M. osloensis *into the shell cavity of the slug host *Deroceras reticulatum *in which the bacteria multiply and kill the slug. As *M. osloensis *is the main killing agent, genes expressed by *M. osloensis *in the slug are likely to play important roles in virulence. Studies on pathogenic interactions between bacteria and lower order hosts are few, but such studies have the potential to shed light on the evolution of bacterial virulence. Therefore, we investigated such an interaction by determining gene expression of *M. osloensis *in its slug host *D. reticulatum *by selectively capturing transcribed sequences.

**Results:**

Thirteen *M. osloensis *genes were identified to be up-regulated post infection in *D. reticulatum*. Compared to the *in vitro *expressed genes in the stationary phase, we found that genes of ubiquinone synthetase (*ubiS*) and acyl-coA synthetase (*acs*) were up-regulated in both *D. reticulatum *and stationary phase *in vitro *cultures, but the remaining 11 genes were exclusively expressed in *D. reticulatum *and are hence infection specific. Mutational analysis on genes of protein-disulfide isomerase (*dsbC*) and *ubiS *showed that the virulence of both mutants to slugs was markedly reduced and could be complemented. Further, compared to the growth rate of wild-type *M. osloensis*, the *dsbC *and *ubiS *mutants showed normal and reduced growth rate *in vitro*, respectively.

**Conclusion:**

We conclude that 11 out of the 13 up-regulated *M. osloensis *genes are infection specific. Distribution of these identified genes in various bacterial pathogens indicates that the virulence genes are conserved among different pathogen-host interactions. Mutagenesis, growth rate and virulence bioassays further confirmed that *ubiS *and *dsbC *genes play important roles in *M. osloensis *survival and virulence, respectively in *D. reticulatum*.

## Background

As the dialog between a host and bacterium requires the coordinated activity of many bacterial gene products in response to the host [1], investigating the bacterial pathogenesis in a diverse set of pathogen-host interactions can contribute to our understanding of the evolution of bacterial virulence. There have been extensive studies on bacteria pathogenic to higher order hosts [2-7], particularly the zebrafish and mouse which have emerged as model animals for the study of bacterial pathogenesis [8]. These studies have provided ample information on the virulence genes essential for pathogenesis, yet we know little about the origin and evolution of these genes. Characterizing the bacterial genes expressed in hosts representing different evolutionary history may enable us to gain a better understanding of the evolution of bacterial virulence [1]. For example, the pathogenic interaction between the nematode *Caenorhabditis elegans *and bacterium *Salmonella enterica *has showed a remarkable overlap between *Salmonella *virulence factors required for human and nematode pathogenesis [9]. While there are several reports of bacterial illness in snakes, tortoises, and reptiles [10-13], little is known about bacteria-involved infections in a slug host [14].

As lower order invertebrates, mollusks are proven to be excellent model systems for studies in neurophysiology, behavioral ecology and population genetics [15]. The slug *Deroceras reticulatum *is one of the important mollusk invertebrates. *Moraxella osloensis *is a gram-negative, oxidase positive, aerobic bacterium within the family Moraxellaceae in the gamma subdivision of the purple bacteria. This bacterium has recently been identified as one of the natural symbionts of a bacteria-feeding nematode, *Phasmarhabditis hermaphrodita *(Rhabditida: Rhabditidae), which is a lethal endoparasite of slugs [16,17], including the slug *D. reticulatum *[18]. In nature, bacteria colonize the gut of nematode infective juveniles (IJs) which represent a specialized stage of development adapted for survival in the unfavorable environment. The IJs seek out and enter the slug's shell cavity through the posterior mantle region. Once inside the shell cavity, the bacteria are released, and the IJs resume growth, feeding on the multiplying bacteria [18-20]. The infected slugs die in 4–10 days, and the nematodes colonize the entire cadaver and produce next generation IJs which leave the cadaver to seek a new host [18].

The lethality of these nematodes to slugs has been shown to correlate with the number of *M. osloensis *cells carried by IJs [20]. Tan and Grewal [20] demonstrated that the 72 h old *M. osloensis *cultures inoculated into the shell cavity were highly pathogenic to the slug. They further reported that *M. osloensis *produced an endotoxin which was identified to be a rough type lipopolysaccharide (LPS) with a molecular weight of 5300 KD, and the purified *M. osloensis *LPS was toxic to the slug with an estimated 50% lethal dose of 48 μg when injected into the shell cavity [21]. Although these studies laid the foundation for the bacteria-slug interaction, the virulence mechanisms of *M. osloensis *that result in pathogenesis and slug mortality are not established. The present study was designed to determine the molecular and genetic basis of *M. osloensis *virulence to the slug *D. reticulatum*.

Several techniques have been developed to study bacterial genes that are expressed during infection or that are required for virulence in the host during infection. The most commonly used techniques include *in vivo *expression technology (IVET) [22], signature-tagged mutagenesis (STM) [23] and differential fluorescence induction (DFI) [24]. Recently, the selective capture of transcribed sequences (SCOTS) technique has been developed to study bacterial gene expression specifically in macrophage [25]. This technique has been used to identify bacterial virulence factors, and has been demonstrated to be capable of identifying genes expressed in specific tissues of infected animals [5,26]. More recently, microarray analysis has also been applied in monitoring bacterial gene expression during host infection [27]. As the host and bacterial cDNAs can be easily differentiated using SCOTS, and this technique does not require prior genetic information of the pathogen, we applied SCOTS to determine *M. osloensis *gene expression in the slug host *D. reticulatum *at two time points following infection. Because important changes in gene expression can occur in bacteria during transition from active growth to stationary phase [5,28], we also investigated differential gene expression of 72 h *M. osloensis *in the *in vitro *cultures (stationary phase) relative to 24 h cultures (log phase) to confirm the infection specificity of the *in vivo *expressed genes. Mutational analyses (virulence of mutants to the slug and *in vitro *growth rate of mutants) were also carried out to examine the roles of selected genes.

## Results

### Differentially *in vivo *expressed genes

**Figure 1:**
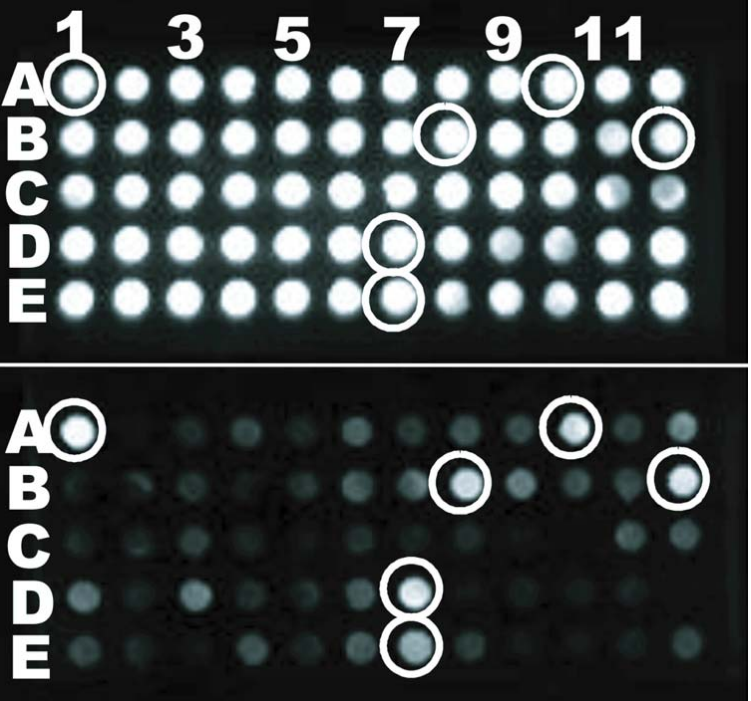
One of the dot blots (exposing 2 min) showing enriched cDNAs hybridized to a digoxigenin-labeled probe generated from normalized *in vivo *cDNAs (upper) or normalized *in vitro *cDNAs (lower) after three rounds of SCOTS normalization. The dots at the same position in the two arrays were loaded with the same amplicon of each individual clone from the enriched cDNA library, and the concentration of probes was standardized to be the same.

To identify *M. osloensis *transcripts specifically expressed in *D. reticulatum*, we injected slugs with 5 × 10^7 ^bacterial cells. We evaluated colonization by analyzing *M. osloensis *cell counts, and found that bacterial counts varied from 10^5 ^to 10^8 ^colony-forming units (CFU) per *D. reticulatum *48 h or 96 h post-inoculation, indicating colonization and persistence of the injected bacteria in the slug. Transcripts expressed by *M. osloensis *in the slug at 48 h and 96 h post inoculation were identified by subjecting the *in vivo *cDNAs to three iterations of SCOTS in the presence of the transcripts expressed by 48 h late log-phase *M. osloensis in vitro *cultured in the Brain Heart Infusion (BHI) (Difco) medium. The enriched cDNAs were cloned into a TA vector. Each individual clone was further screened by dot blot hybridization (Fig. 1). The individual clones with signals stronger than or present on the blot hybridized to the *in vivo *cDNAs compared to that with signals on the blot hybridized to *in vitro *cDNAs were chosen for nucleotide sequencing and analysis. A total of 97 clones (27 from 48 h post inoculation, and 70 from 96 h post inoculation) were sequenced. These screened cDNAs represented the differentially expressed genes within the slug but in lower abundance or absent in the 48 h *in vitro *cultures. A fraction of the identified sequences was further confirmed to be *M. osloensis *specific by PCR amplification of *M. osloensis *genomic DNA with the designed primers based on identified sequences, and the results confirmed that *M. osloensis *transcripts were successfully isolated from the infected slugs using the SCOTS technique. The identified sequences were analyzed using the non redundant algorithms of BLAST [29] in the website of NCBI (National Center for Biotechnology Information) [30]. For each identified sequence, about two to five cDNA clones were detected in the screened library, and these clones showed the same hits in the GenBank. In this study, the identity of up-regulated sequences at 48 h and 96 h post infection was identical. We identified 13 distinct sequences (arbitrarily termed as *M1*, *M2*, *M3*...*M13*) representing 13 up-regulated genes (Table 1) which carry putative functions in cell structure integrity, energy metabolism, degradation, and translocation. Three of these sequences, *M11*, *M12*, and *M13*, have similarity with genes encoding hypothetical proteins, and further analysis by inverse PCR amplification and sequencing demonstrated that they were similar to the genes encoding preprotein translocase (SecA), acyl-coA synthetases (Acs), and acetyl-coA carboxylase (Acc). One of the sequences (*M3*) did not exhibit similarity to any genes or gene products in current databases, and is possibly novel. *M1*, *M2 *and *M4 *genes contain translated sequences of "Gly-Asp-Pro-Asp" repeats, and are similar to membrane proteins. Sequence *M5 *shows similarity with the gene encoding protein-disulfide isomerase (DsbC) in other bacteria. Sequences *M6*, *M8*, and *M10 *have similarity to genes that encode iron regulation related proteins in other bacteria; thus, the identification of these genes suggests that iron availability in slug host may be limited. The sequence *M7 *shares similarity to the gene encoding acetyltransferase (Ats) that functions in the energy metabolism and is often involved in antigen synthesis [31]. The sequence *M9 *has similarity with the predicted gene β-carboxymuconolactone decarboxylase (PCA) which encodes enzymes participating in the conversion of protocatechuate to succinate and acetylcoenzyme A (Acyl-CoA) [32].

**Table 1:** SCOTS identified *M. osloensis *genes differentially expressed *in vivo*

Seq	Gene	Accession	%Identity/%Similarity^a^	E-value	Span^b^	Possible function^c^
*M1*	*sclB*	DQ324274	50/63, *Streptococcus*	9e-24	96	Surface lipoprotein
*M2*	*vspC*	DQ324276	46/56, *Mycobacterium*	4e-06	50	Ala and Pro rich protein
*M11*	*secA*	DQ324262	77/88, *Psychrobacter*	5e-127	299	Preprotein translocase
*M12*	*acs*	DQ324263	76/89, *Psychrobacter*	0	442	Acyl-CoA synthetases
*M13*	*acc*	DQ324264	85/90, *Psychrobacter*	0	480	Acetyl-CoA carboxylase
*M4*	*spp*	DQ324275	50/63, *Lactobacillus*	2e-05	37	Surface protein precursor
*M5*	*dsbC*	DQ324260	49/67, *Psychrobacter*	5e-70	257	Disulfide isomerase
*M6*	*abp*	DQ324271	80/86, *Acinetobacter*	2e-51	145	ATP-binding protein
*M7*	*ats*	DQ324266	31/46, *Psychrobacter*	7e-11	163	Acetyltransferases
*M8*	*ubiS*	DQ324261	71/83, *Psychrobacter*	0	557	Ubiquinone synthesis
*M9*	*pca*	DQ324272	67/80, *Psychrobacter*	3e-46	131	Carboxymuconolactone decarboxylase
*M10*	*adh*	DQ324273	90/96, *Acinetobacter*	5e-55	114	Aldehyde dehydrogenase
*M3*			No similarity			Unknown

^a ^%Identity/%Similarity percentages apply to amino acids.

^b ^Span means the number of amino acids over which similarity was detected.

^c ^Genes with highest similarity to SCOTS identified sequences.

### Differentially *in vitro *expressed genes at the stationary phase

In order to determine if the identified *in vivo *expressed genes are infection-specific, the differential expression of transcripts corresponding to 72 h *M. osloensis *cultures (stationary phase, data not shown) relative to 24 h cultures (early-log phase) was examined by SCOTS technique. Nine sequences (descriptive *Mo1*, *Mo2*...*Mo9*) were identified to be differentially expressed in 72 h relative to 24 h cultures (Table 2). One sequence (*Mo9*) did not show any similarity in current NCBI databases, and is thus possibly novel for *M. osloensis*. Sequences *Mo2 *and *Mo4 *have similarity to transposases and integrase, *Mo6 *is similar to topoisomerase, *Mo1 *has high similarity to Actin-like ATPase, and *Mo7 *is similar to ATPase involved in DNA replication. Two sequences, *Mo3 *and *Mo8 *are similar to genes of *ubiS *and *acs *that were also found to be differentially expressed under *in vivo *condition, suggesting that these two genes are not infection specific.

**Table 2:** SCOTS identified genes exclusively expressed by 72 h *in vitro *cultures

Seq	Gene	Accession	%Identity/similarity^a^	E-value	Span^b^	Possible function^c^
*Mo1*	*ala*	DQ904630	87/94, *Flavobacterium*	1e-27	86	Actin-like ATPase
*Mo2*	*tra*	DQ904631	72/78, *Psychrobacter*	6e-13	52	Transposase
*Mo3*	*ubiS*	DQ904632	97/100, *Moraxella*	1e-12	47	Ubiquinone synthesis
*Mo4*	*int*	DQ904633	75/84, *Psychrobacter*	3e-21	105	Integrase
*Mo5*		DQ324278	80/90, *Psychrobacter*	1e-46	110	Conserved protein
*Mo6*	*top*	DQ324269	71/88, *Psychrobacter*	2e-28	80	Topoisomerase
*Mo7*	*pol*	DQ904634	38/58, *Psychrobacter*	4e-17	134	DNA polymerase
*Mo8*	*acs*	DQ904635	64/82, *Acinetobacter*	3e-24	79	Acyl-CoA synthetase
*Mo9*			No similarity			Unknown

^a ^%Identity/%Similarity percentages apply to amino acids.

^b ^Span means the number of amino acids over which similarity was detected.

^c ^Genes with highest similarity to SCOTS identified sequences.

### Mutational analysis of identified genes

**Figure 2:**
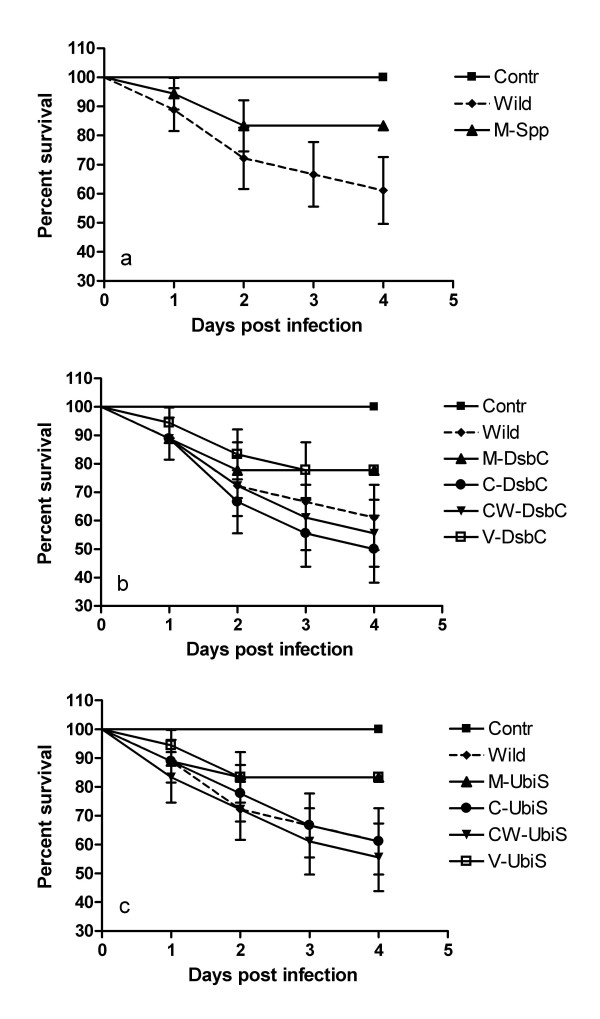
The slug survival post *M. osloensis *infection was plotted with Kaplan-Meier plots. The slug mortality caused by bacterial cultures is significantly different between wild type and mutants post 2-days infection. Contr, Saline; Wild, wild-type *M. osloensis*; M-Spp, mutant of *spp *gene; M-DsbC, mutant of *dsbC *gene; V-DsbC, *dsbC *mutant containing the empty vector; C-DsbC, the complemented *dsbC *mutant; CW-DsbC, wild-type *M. osloensis *containing the plasmid borne *dsbC *gene (CW-dsbC serves as a control for C-dsbC); M-UbiS, mutants of *ubiS *gene; V-UbiS, *ubiS *mutant containing the empty vector; C-UbiS, the complemented *ubiS *mutant; CW-UbiS, wild-type *M. osloensis *containing the plasmid borne *ubiS *gene (CW-ubiS serves as a control for C-ubiS).

To investigate the role of up-regulated genes in *M. osloensis *in the slug, three mutants of selected genes were constructed using inverse PCR combined with insertional inactivation. The selected genes represent three distinct groups: cell surface protein (*M4*, *spp*) which has been previously reported as a virulence gene in pathogenic bacteria [33,34], protein-disulfide isomerase (*M5*, *dsbC*) which has been recently identified as a virulence gene in pathogenic bacteria associated with higher order hosts [35], and ubiquinone synthetase (*M8*, *ubiS*) which has been reported to be important for bacterial survival and was found to be up-regulated *in vitro *at the stationary phase [36,37]. As described in Materials and Methods, the inverse PCR was performed using primers with the engineered *Ape *I and *Bma *I sites. The 800-bp non-polar kanamycin resistance gene was inserted into the 50-bp internal deletion within the open reading frame (ORF) of *spp*, *dsbC*, or *ubiS*. Compared to the whole sequences of *dsbC *and *ubiS *amplified in this study, the insertions were located 250 bp downstream from the ATG codon of *dsbC*, and 300 bp downstream from the GTG start codon for *ubiS*. After natural transformation and double recombination, the resulting isogenic mutants were obtained *in vitro *after 72 h incubation, and they were termed as *M-spp*, *M-dsbC*, and *M-ubiS*, respectively. The virulence of wild type and mutants was determined by direct inoculation into the slug *D. reticulatum *and subsequent survival analysis. All three mutants demonstrated the level of virulence similar to the wild-type strain in the first two days (*P *> 0.05) (Fig. 2). However, at 3 days post infection, all three mutants produced significantly lower slug mortality relative to the wild-type parent strain (*P *= 0.036, 0.015, and 0.023 for *M-spp*, *M-dsbC*, and *M-ubiS*, respectively).

We further complemented the constructed mutants. The full length and promoter region of *dsbC *and *ubiS *were obtained by inverse PCR strategy with the aid of six pairs of primers (data not shown) and six restriction enzymes (*Ape *I, *EcoR *I, *Hinf *I, *Pst *I, *Bma *I, and *Bxa *I).

Amplification of the *spp *ORF was not successful due to the presence of repetitive sequences, and thus was not used for further analysis. The ORF of *dsbC *and *ubiS *were 843 bp and 1671 bp, and the blast searches confirmed that they were similar to protein-disulfide isomerase and ubiquinone synthesis genes, respectively. The sequence analysis revealed that *dsbC *gene had an N-terminal signal peptide, and contained thioredoxin-like domain with the characteristic Cys-x-x-Cys active site. The complemented mutants, termed as *C-dsbC *and *C-ubiS*, were obtained by transferring intact *dsbC *and *ubiS *genes borne in the plasmid into the respective *M. osloensis *mutant by electroporation. The level of virulence of complemented mutants in the slug was similar to wild-type strains (Fig. 2).

### Growth rates of mutants

**Figure 3:**
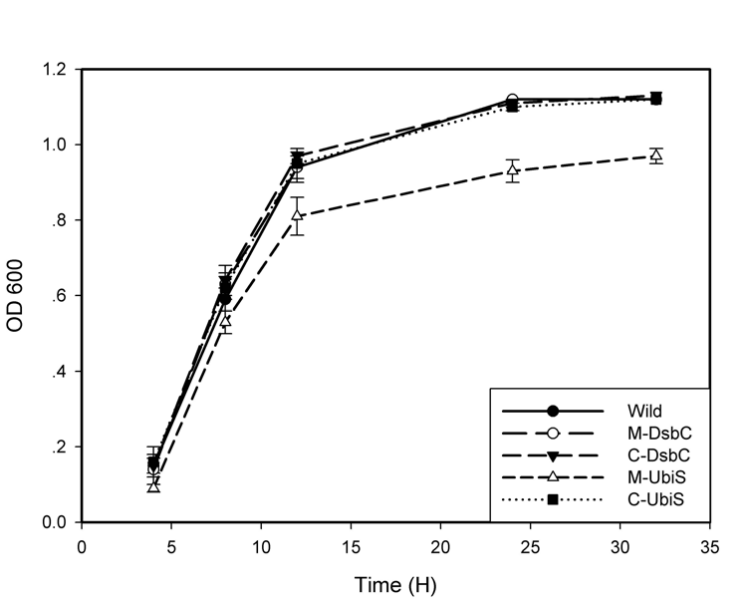
Growth curve for *M. osloensis *in culture derived from measurements of OD 600 over time. Wild: wild-type *M. osloensis*; M-DsbC: mutant of *dsbC *gene; C-DsbC: complemented *dsbC *mutant; M-UbiS: mutant of *ubiS *gene; C-UbiS: complemented *ubiS *mutant. All data are shown as the mean of three repeated experiments with standard errors.

As reduced virulence of mutants may result from growth defect, the growth rates of *dsbC *and *ubiS *mutants in the BHI medium were examined. Compared to the wild-type *M. osloensis*, the *dsbC *mutant did not show any growth defect *in vitro *(Fig. 3). However, the *ubiS *mutant showed significant reduction in growth rate *in vitro*. The *ubiS *mutant grew slower than the wild type and its viability (shown as OD600) was about 12% lower relative to the wild-type strain after 12 h (Fig. 3), and the growth defect became evident at the time of the late log phase entering into the stationary phase. In addition, the growth defect phenotype was fully complemented by the trans-complementation with the gene itself.

## Discussion

The current study is the first description of virulence genes expressed by a bacterium in a slug host. The SCOTS analysis demonstrates that *M. osloensis *expressed 13 genes in the slug host when compared with log-phase bacteria growing *in vitro *in the BHI medium. Comparison with differentially expressed genes by the 72 h relative to 24 h *in vitro *cultures revealed that 11 of the 13 genes appeared infection specific. Among these infection-specific genes, some genes likely play roles in energy metabolism, and may affect virulence indirectly by sustaining the basic cell functions during infection. For example, the *pca *gene identified here plays a role both in nutrition and detoxification in other bacteria [38]. Gene *abp *identified in this study is similar to the genes encoding ATP binding proteins that confer multiple resistance and pH adaptation. Blast search reveals that *abp *also shares high similarity to the polysaccharide export protein gene. In case of pathogenic bacteria, polysaccharide is often secreted as an evolutionary adaptation to adhere to host surfaces and to escape from drying and host immune defense [39,40]. Therefore, it is possible that *abp *gene product may play an important role in *M. osloensis *survival in the slug.

*ubiS *gene exhibits sequence similarity to the gene encoding protein that functions in ubiquinone synthesis. Ubiquinone is a central component of the electron transport chain under aerobic conditions and functions in the formation of disulfide bonds in periplasmic proteins by facilitating the reoxidation of protein-disulfide isomerase [36,37]. As *ubiS *gene was also up-regulated in the stationary phase in *M. osloensis*, this gene may be important for bacterial survival during starvation and could be induced by the stress conditions. Further examination of *ubiS *mutant growth *in vitro *indicated that the reduced virulence of this mutant may be due to the reduced growth rate.

Preproteins are exported primarily across the cytoplasmic membrane to the periplasm or the outer membrane by the sec system composed of the translocase SecYEG (a protein-conducting channel) and SecA (an ATP-dependent motor protein) proteins [41-43]. SecA interacts dynamically with the SecYEG components to drive the transmembrane movement of newly synthesized preproteins [44]. Tomkiewicz et al. [45] showed that in *Escherichia coli *SecA drives a constant rate of preprotein translocation consistent with a stepping mechanism of translocation. Further, the *secA *expression is subject to the translational control in response to the cellular activity of protein translocation [44,46]. Therefore, the up-regulation of *secA *gene *in vivo *in *M. osloensis *suggests that the expression of *secA *is regulated by the infection process.

Three of the identified genes, *sclB*, *vspC*, and *spp*, are similar to the genes encoding structural proteins of the outer membrane. Outer membrane proteins are known to be critical for establishment of disease in the host by causing resistance to immune killing [33,47] as demonstrated in a fish pathogenic bacterium, *Edwardsiella tarda *[34]. Virulence of *spp *mutant to the slug is significantly reduced indicating that surface membrane proteins are also important for *M. osloensis *infection in the slug.

Genes *ats*, *adh*, *acs *and *acc *found to be up-regulated in vivo in *M. osloensis *are similar to the genes reported to be important for the virulence in other bacterial pathogens. *ats *gene is similar to the gene for acetyltransferase which has been known as an effector protein in bacterium *Yersinia *[48], and plays an important role in regulating biological signaling. *ald *gene is similar to the gene for aldehyde dehydrogenase (AldH). In *Vibrio cholerae*, *aldH *gene has been found to be located in a pathogenicity island in epidemic and pandemic strains but absent from non-pathogenic strains [49]. Gene *acs *shares sequence similarity with the gene of long chain fatty acid CoA synthetase (FadD), an enzyme involved in lipid synthesis and whose expression may be important at various stages of infection [50-53]. *fadD *gene expression has been demonstrated to be important in virulence in a number of organisms, including *Xanthomonas campestris *[54] and *Salmonella enterica *serovar Typhimurium [55]. In this study, *acs *gene was identified to be up-regulated both *in vivo *and *in vitro *stationary phase. Thus, the expression of *acs *protein may be related to *M. osloensis *survival and virulence under *in vivo *conditions.

The gene *dsbC *belongs to the gene family *dsb *involved in disulfide bond exchange which catalyses the folding of various factors including virulence determinants such as the components of type III secretory machinery and assembly of type II secreted subunits into effector proteins in a number of bacteria [56-58]. Various gram-negative bacterial pathogens use type II or III secretion system as a basic virulence mechanism [2]. Dsb protein has been identified as a virulence factor in diverse bacterial pathogens associated with different higher order hosts. In pathogenic *Shigella flexneri*, *dsb *gene is necessary for intracellular survival and cell-to-cell spread in the host [57]. In the fish pathogenic bacterium, *Flavobacterium psychrophilum*, mutants of Dsb-like protein gene exhibited reduced virulence and cytotoxicity [59]. *dsb *gene was also identified as a virulence factor by SCOTS technique in the pig pathogenic bacterium *Actinobacillus pleuropneumoniae *[30]. Mutation of *dsbC *in *Bordetella pertussis *resulted in decreased toxin secretion [60]. In our study, the *dsbC *mutant showed significantly reduced virulence to the slug, but its normal growth rate *in vitro *confirms that *dsbC *serves as a virulence gene in *M. osloensis *in the slug host.

Previous studies by Tan and Grewal [21] showed that lipopolysaccharide (LPS) of *M. osloensis *alone was sufficient to cause slug mortality. Compared to the virulence defects of *dsbC *and *ubiS *mutant, it may be hypothesized that there is relationship between LPS and DsbC or UbiS despite the current lack of experimental evidence. The link between UbiS and Dsb has been reported in a study in *Escherichia coli *which demonstrated that disulfide bond formation in exported proteins is catalyzed by Dsb protein, and is directly coupled to the electron transport chain via reoxidation of Dsb protein by either ubiquinone or menaquinone [35]. In addition, most animals possess agglutinating activity to recognize self or non-self particles, and the agglutinins are believed to be lectins in invertebrates. According to Barker [15], the land slug *Incilaria *contains three lectins which show significant sequence similarity to other animals, having two disulphide bonds which may be related to lectin activity. Since *dsb *gene family in prokaryotes has the ability to rearrange non-native disulfides to their native configuration [61], it is proposed that *dsbC *functioning as a virulence factor in *M. osloensis *may be related to the host self/non-self recognition.

## Conclusion

In this study, no differences in *M. osloensis *gene expression between 48 h and 96 h post inoculation were observed indicating that the infection was established in 48 h. Thirteen *M. osloensis *genes were up-regulated in the slug. Most of these genes have been previously identified in other bacterial pathogens, suggesting the conserved nature of bacterial virulence genes. Among the 13 up-regulated genes, *acs *and *ubiS *were also expressed in the bacterial stationary phase *in vitro*, and *ubiS *was further confirmed to be important for *M. osloensis *survival in the slug. The remaining 11 genes were infection specific. One of these genes, *dsbC*, belonging to *dsb *gene family, was confirmed to be a virulence gene in *M. osloensis *based on the mutagenesis study, growth rate and virulence bioassays. To our knowledge this is the first report on bacterial gene expression in a slug host interface.

## Methods

### Bacteria, slugs, and culture conditions

A type strain of *Moraxella osloensis *acquired from the American Type Culture Collection (ATCC) was used in this study. The bacteria were cultured in BHI (Difco) broth at 25°C, and confirmed to be *M. osloensis *by 16S rDNA sequencing. The association between this bacterial strain and the nematode *P. hermaphrodita *had been previously evaluated by assessing the recovery of infective juveniles in the bacterial cultures. The growth rate of *M. osloensis *was determined by turbidity measurements according to Tortora et al. [28]. Slug *D. reticulatum *adults were collected from the field and fed on pieces of fresh carrots at 18°C for at least 12 days. All the slugs were fed on sterile water for three days to wash the intestine before bacterial infection. Only healthy actively moving adult slugs were used in experiments.

### General techniques

Bacterial genomic DNA was prepared using standard method for Gram negative bacteria [62]. Biotinylation of bacterial genomic DNA was obtained with EZ-Link Psoralen-PEO-Biotin (Pierce) according to the manufacturer's instructions. The total RNA was isolated using TRIzol reagent (Invitrogen) according to the manufacturer's instructions. RNA samples were treated with RNase-free DNase I (Ambion, Austin, TX) according to the manufacturer's guidelines, and were concentrated by spectrophotometer and gel electrophoresis. Total RNA was converted to first-strand cDNA using Superscript II reverse transcriptase (Invitrogen RT-PCR kit) according to the manufacturer's instructions. First strand cDNA was made double-stranded with Klenow fragment (NEB, Beverly, MA) as described by Froussard [63]. Restriction endonucleases and ligase enzymes (Promega) were used according to the manufacturer's guidelines. The DNA samples were sequenced at the Biotechnology Center, Madison, WI, USA; and McGill University and Genome Quebec Innovation Centre, Montreal, Canada. Sequence analysis was carried out using BLAST algorithms (blastx) in GenBank NCBI.

### SCOTS analysis

We extended SCOTS technique to determine *in vivo *gene expression of *M. osloensis *within the slug at two different time points post infection. The SCOTS technique for this study involves the capture of *in vivo *transcribed sequences by using biotinylated *M. osloensis *genomic DNA coupled to streptavidin-coated paramagnetic beads and a PCR-based subtractive hybridization with transcripts from the *in vitro *cultured bacteria.

Briefly, for the control, 5 μg total RNA samples obtained from 48 h *M. osloensis *cultures (OD 600 = 0.8) were converted to cDNAs and then made double-stranded. The primer (SCOT09) with a defined sequence (SCOT0) at the 5' end and random sequence at the 3' end was used for both first and second strand cDNA synthesis (Table 3). cDNAs were then PCR amplified using the defined primer (SCOT0) for 30 cycles. Bacterial cDNA normalization was done according to Graham and Clark-Curtiss with some modifications [25]. In this study, *M. osloensis *ribosomal operon (rDNA) was amplified using primers Mor-F and Mor-R (Table 3). A plasmid containing the amplified operon was constructed using TOPO XL PCR Cloning kit (Invitrogen), and the constructed plasmid was used to block the abundant rDNA sequence in order to effectively capture the cDNA molecules representing mRNA transcripts. The rDNA operon was added to biotinylated genomic DNA at a ratio of 10:1. The genomic DNA-rDNA mixture was sonicated to a size range of 1 to 5 kb. The sonicated, biotinylated genomic DNA-rDNA mixture containing 6 μg rDNA and 0.6 μg genomic DNA was denatured and hybridized for 30 min at 67°C. PCR amplified cDNAs (6 μg) from 48 h cultures were denatured and added to the genomic DNA-rDNA prehybridized mixture, and hybridized at 67°C for 24 h. Streptavidin magnesphere paramagnetic particles (Promega) were used to capture the bacterial cDNA that hybridized to biotinylated genomic DNA according to the manufacturer's instructions. Captured cDNAs were then eluted, precipitated, and amplified by PCR with the defined primers for additional two successive rounds of SCOTS. The finally amplified cDNAs were considered to be normalized *in vitro *cDNAs.

**Table 3:** Primers used in SCOTS analysis

Primers	Sequences
Mor-F	5-GAACGCTGGCGGCAGGCTTAACACATGC
Mor-R	5-GCTGGCGATGACTTACTCTCACATGGCTAACGCC
SCOT0	5-ATCCACCTATCCCAGTAGGAG
SCOT09	5-ATCCACCTATCCCAGTAGGAGNNNNNNNNN-3
SCOT18	5-GACAGATTCGCACTTAACCCT
SCOT189	5-GACAGATTCGCACTTAACCCTNNNNNNNNN-3
SCOT110	5-ATGCGAATCCAGACTGTAAGA
SCOT1109	5-ATGCGAATCCAGACTGTAAGANNNNNNNNN-3

For the treatment, 50 μl suspension of 48 h *M. osloensis *cultures (OD 600 = 0.8) containing 5 × 10^7 ^cells according to the bioassay test [24] was inoculated into the shell cavity of slug *D. reticulatum*. Inoculation into the shell cavity is mimicking the natural infection of the slug by nematode-bacteria complex. Before RNA isolation, a parallel experiment was performed to evaluate the number of viable *M. osloensis *to confirm the successful bacterial infection in the slug post inoculation. In brief, the infected slug was surface sterilized by immersing in 0.1% thimerosal for 3 h, washed by sterile water, and mortared. The slug homogenate was serially (10-fold) diluted and plated on Mueller-Hinton II agar (MHAII; BBL Becton Dickinson, Cockeysville, Md). The number of CFU was then counted based on the colony color and oxidase reaction [64]. After that, the total RNA was isolated from survived slugs. As about 50% slug mortality occurred within 96 h post bacterial injection or nematode infection, two time point, 48 and 96 h post bacterial injection were selected for total RNA isolation. Three 5 μg total RNA samples obtained from three infected slugs were pooled and made double-stranded by the random primer (Primer SCOT189 was used for 48 h post infection, and SCOT1109 was used for 96 h post infection). Double-stranded cDNAs were then PCR amplified for 30 cycles by the defined primer (Primer SCOT18 was used for post 48 h infection, and SCOT110 was used for post 96 h infection). Amplified cDNAs from 48 h or 94 h post infection were added to the genomic DNA-rDNA prehybridized mixture to hybridize at 67°C for 24 h. The bacterial cDNAs were then captured by streptavidin-coated magnetic particles. The captured cDNAs were eluted and PCR amplified. In the first round of SCOTS, three separate samples of cDNAs were captured by hybridization to biotinylated genomic DNA in parallel reactions. After the first round of SCOTS, the three amplified cDNA preparations for 48 h or 96 h post infection were combined, denatured, and again hybridized to genomic DNA-rDNA mixture for two successive rounds of SCOTS. The finally amplified cDNAs were considered to be normalized *in vivo *cDNAs.

To enrich the normalized *in vivo *cDNAs, the normalized cDNAs from 48 h or 96 h post infection were hybridized to biotinylated genomic DNA that has been prehybridized with both rDNA and normalized *in vitro *cDNAs. After hybridization, the bacterial cDNAs were captured and PCR amplified for next round of enrichment. Finally, the enriched bacterial cDNAs were cloned into an original TA cloning kit (Invitrogen). Each individual clone was PCR amplified, and the concentration of the amplicon was standardized to 0.3 μg/μl. The 80 μl individual amplicon was transferred to each well of the dot-blot containing the positively charged nylon membrane using the low vacuum. The nylon membrane with immobilized individual cDNA was transferred into a hybridization bottle for hybridization using Dig easy hyb granules (Roche) according to the manufacturer's instruction. Digoxigenin-labeled probes were made from the normalized *in vitro *or *in vivo *cDNAs using PCR Dig Probe Synthesis kit from Roche Molecular Biochemicals (Indianapolis, Ind.) in accordance with the manufacturer's instructions. The concentration of the generated probes was standardized to be same. Individual cDNA was then screened by dot blot hybridization with the generated probe. The hybridization between the probe and the individual cDNA was performed at 65°C for 24 h. The dilution of anti-digoxigenin-HRP conjugate (1: 800) and Amersham ECL Plus western blotting detection reagents (GE healthcare Bio-Sciences Corp, Piscataway, NJ) were used to detect the hybridization between individual cDNA and the probe. The individual clones with signals stronger or present on the blot hybridized to the normalized *in vivo *cDNAs compared to that with signals on the blot hybridized to the normalized *in vitro *cDNAs were chosen for nucleotide sequencing and analysis.

Furthermore, in order to determine that the identified *in vivo *expressed genes were specific to infection or suppressive condition, we examined the differential *in vitro *gene expression of 72 h *M. osloensis *cultures (stationary phase) relative to 24 h cultures (early-log phase) using the similar procedure.

### Inactivation of SCOTS identified genes

Several *M. osloensis *mutants of SCOTS identified genes were constructed by insertion-deletion strategy using inverse PCR according to Furano and Campagnar [65]. Briefly, the SCOTS identified cDNA molecule was cloned into a PGEM-T vector (Promega). The primers with engineered restriction sites were used in the inverse PCR to amplify the cDNA fragment. This resulted in a deletion of about 50 bp nucleotides internal to SCOTS identified sequence. The nonpolar kanamycin resistance gene was amplified from a TA vector with the engineered primers. Inverse PCR product and kanamycin resistance gene were subjected to restriction digestion and ligation, resulting in the plasmid containing the cDNA sequence and kanamycin resistance gene. Sequence analysis was performed to confirm the proper insertion of the cassette. The resulting plasmid was PCR amplified, and product was used to naturally transform wild-type *M. osloensis *[65,66]. Insertion of the kanamycin gene through a double recombination resulted in the disruption of the wild-type gene. In brief, a 100 μl aliquot of an early-log phase *M. osloensis *culture was plated onto BHI agar, and 20 ng of the purified mutagenesis constructed sequence from above was spotted onto a portion of the bacterial lawn. After 6 h incubation under standard growth conditions, the area of the bacterial lawn that had been inoculated with the constructed sequence was swabbed onto BHI agar plates containing kanamycin or added to BHI broth (broth method) containing kanamycin and slug homogenate. Samples from broth method were incubated for 12 h, and then different dilutions were spot inoculated on the selective plates containing kanamycin to select the mutants. The mutant chromosomal DNA was isolated and subjected to PCR analysis as well as sequence analysis to confirm the integration of the inactivated genes into the genome. The stability of these mutants was verified by growing the mutants in medium lacking kanamycin and the frequency of kanamycin resistance, and the mutants with stable gene disruptions were selected for the virulence test and complementation study.

Due to the limited genetic information for *M. osloensis*, we used inverse PCR strategy to amplify the full sequence of SCOTS captured sequences. The amplified sequences were further analyzed by the bio-software such as SignalP 3.0 [67]. The cloned intact genes were cloned into pCR2.1 using the TA Cloning kit (Invitrogen) according to the manufacturer's instructions. The plasmid containing the intact genes was then transferred into the responded mutant and wild-type backgrounds by electroporation. The complemented mutants were further confirmed before virulence test. The virulence of wild type, mutants, and complemented mutants of *M. osloensis *cultures were determined by quantifying slug mortality following the injection of 72 h bacterial culture into the shell cavity as described below.

### Virulence of *M. osloensis *to the slug

The virulence of *M. osloensis *cultures was determined by quantifying slug mortality following the injection of bacteria into the shell cavity. The different bacterial cells were precipitated and washed several times using a sterile saline solution (0.85% NaCl), and the total numbers of bacteria in each suspension were measured with a spectrophotometer at a wavelength 600 nm. Different *M. osloensis *cultures were injected into the slug shell cavity with the same dose used in the SCOTS analysis, and which is consistent with the dose used by Tan and Grewal [20]. Slugs injected with the saline solution served as controls. Six slugs were tested in each treatment, and three replicas were used for each treatment. Slug mortality was recorded 4 days after inoculation at 18°C. The survival analysis was plotted as Kaplan-Meier plots by using the statistical software GraphPad Prism 4 (GraphPad software Inc.), and the Chi-square value for significance at each time point was calculated with significant difference tests at P= 0.05.

### Growth rate of mutants in the media

*M. osloensis *cells of wild type, mutants, and complemented mutants were streaked on BHI agar plates and incubated at 25°C for 72 h. Single colonies from the cultures were picked and inoculated into 3 ml BHI broth, and cultured at 25°Cfor 72 h. To obtain growth rate, 100 ml BHI broth was inoculated with 1 ml of 72 h cultured bacterial cells, and the OD 600 value was recorded until the wild-type bacteria reached the stationary phase.

## List of abbreviations

IJs, infective juveniles; SCOTS, selective capture of transcribed sequences

## Authors' contributions

RA performed all the experiments, analyzed the data, and wrote the first draft. Both SS and PSG designed the study, wrote the grant proposal and obtained the funding. PSG initiated the project and helped in writing the manuscript. All authors read and approved the final manuscript.
